# The Dark Side of Grasslands: Endophyte Toxicosis in Horses—Exposure Risks, Health Consequences, and Management

**DOI:** 10.3390/toxins18030117

**Published:** 2026-02-24

**Authors:** Qendrim Zebeli, Lena Lindner, Barbara U. Metzler-Zebeli

**Affiliations:** Centre for Animal Nutrition and Welfare, Clinical Department for Farm Animals and Food System Science, University of Veterinary Medicine Vienna, 1210 Vienna, Austriabarbara.metzler@vetmeduni.ac.at (B.U.M.-Z.)

**Keywords:** fescue toxicosis, ergot alkaloids, equine health

## Abstract

Grasslands are the cornerstone of horse feeding, used for grazing and to produce roughages and their products. However, improper grassland management hides several threats for equine health. In this context, grasslands contaminated with toxin-producing endophytes are considered an increasing threat for horses in many parts of the world. Endophytes are fungi that may grow in a mutualistic relationship in a range of grasses, including fescue grass and perennial ryegrass, two foliage species commonly found in European and American meadows and pastures. The endophytes produce alkaloids that are highly toxic to insects and animals, including horses. Among the four types of endophyte alkaloids, namely peramine, loline, indole diterpene, and ergot alkaloids, the latter two are known to be (neuro)toxic to horses. Recent research indicates that increasing concentrations and co-occurrence of ergot alkaloids and indole diterpene in horse pastures and meadows, especially during hot and arid summer months, increase the risk of endophyte toxicosis in horses. The main aim of this review article is to summarize the most recent knowledge on endophytic alkaloids of grasslands and products thereof, and the resulting endophyte toxicosis in horses, focusing mainly on the exposure risks, symptoms and management strategies.

## 1. Introduction

Grasslands, including pastures and meadows, are essential for horse feeding and welfare. Pastures are commonly used for grazing, whereas meadows are used for producing preserved roughages such as hay, haylage, grass silage; all these roughage feeds being the most important part of the equine diet. Roughages are the main source of energy and nutrients in the equine diet, including structural fiber, which is needed for dental and gastrointestinal health. Although grasslands are seen as the natural equine habitat, consumption of foliage contaminated with various poisonous plants and biotoxins constitutes a substantial threat for horse health [1,2]. Besides being used to produce roughages, meadows are also used to produce various processed roughage products such as hay cobs and cubes, which are widely used to supplement the equine diet. New studies suggest that processed roughage products including hay cobs, cubes or grain-free muesli products may also be a source of several biotoxins, amplifying the risk to equine health [3]. In particular, the grasslands contaminated with toxin-producing endophytes are considered an increasing threat in many parts of the world, including Europe. Illnesses caused by the consumption of endophyte-contaminated feed are referred to as “endophyte toxicity”, which is a widely known phenomenon in the USA, Australia, and New Zealand [2], but has become a concern on the European continent as well [1,2]. Diseased livestock and horses linked to the consumption of grass or hay originating from grasslands contaminated with toxin-producing endophytes have been reported in Germany, Belgium, the Netherlands, France, and the United Kingdom [2].

Endophytes are fungi that may grow within a range of grasses from the *Pooideae* family, including fescue grass and perennial ryegrass [1]. These cool-season perennial grasses are well adapted to their surrounding environment and climate, combining several beneficial traits including high nutritional value and weather resistance and making up a large percentage of the foliage species on European and American meadows and pastures [4]. Common subspecies of the fescue variety include the subspecies *Festuca arundinacea* (also known as *Schedonorus arundinaceus* or *Lolium arundinaceum*), *Festuca pratensis*, and *Festuca rubra*. Subspecies of the perennial ryegrass family are *Lolium perenne* and *Lolium multiflorum*. Of particular relevance for equine grasslands are the endophyte species *Epichloë* (formerly known as *Neotyphodium* or *Acremonium* spp.), including *E. coenophiala* syn *N. coenophialum*, and *E. festucea var. lolii* syn *N. lolii* [5], which infest fescue grass and perennial ryegrass and are the main threats for endophyte toxicity in horses.

The main aim of this review article is to summarize the most recent knowledge on the occurrence of endophytes and endophytic alkaloids of grasslands and products thereof and their resulting endophyte toxicosis in horses, focusing mainly on the exposure risks, symptoms and management strategies.

## 2. Endophytes and Their Alkaloids Relevant for Equine Toxicosis

While some endophyte species are visible to the naked eye, such as *Claviceps purpurea* in grains (rye, wheat and barley), some species can only be detected microscopically or through molecular biological laboratory examination, such as the above-mentioned subspecies of *Epichloë* [1,2]. Endophytes form a symbiotic relationship with their host plant, so that both the fungus and the plant draw benefits from each other. The fungus retains nutrients from the plant, which enables its growth and reproduction. At the same time, the fungus produces organic compounds—also known as alkaloids—that aid the growth of the plant and protect it from predators (i.e., insects and herbivorous mammals) and environmental stressors, such as lack of water and extended drought [1,2]. Indeed, grasses not infected by endophytes have shown to be less resistant to biotic stress factors, such as insects and nematodes, and abiotic stress factors like environmental change [6]. Although the endophyte alkaloids are beneficial to the plant, they also elicit strong toxicity, being harmful to mammals including horses. In general, there are four types of alkaloids produced by *Epichloë* endophytes, namely peramine, loline, indole diterpene, and ergot alkaloids. The first two classes primarily affect insects, whereas the latter two are known to be (neuro)toxic to mammals as well [7,8].

Table 1 provides an overview of the most common endophytes found in grasses, complete with the alkaloids produced by the endophytes and the illnesses they may cause in horses when ingested. Among the alkaloids, the indole-diterpenoids (specifically lolitrem B and its precursor paxilline) and ergot alkaloids found in tall fescue grass and perennial ryegrass severely affect equine health. Chemically, ergot alkaloids are composed primarily of the ergopeptine alkaloids (ergovaline, ergotamine, ergocristine, ergosine, ergocryptine, ergocornine), and the ergoline alkaloids (lysergic acid, lysergol, lysergic acid amide, and ergonovine). Ergovaline is considered to be the most toxic of the ergopeptine alkaloids [1,9] and is the most researched alkaloid in horses. Ergonovine and lysergic acid amide are reported for their dose-dependent cytotoxic effects on animal smooth muscle cells [10].

**Figure 1:**
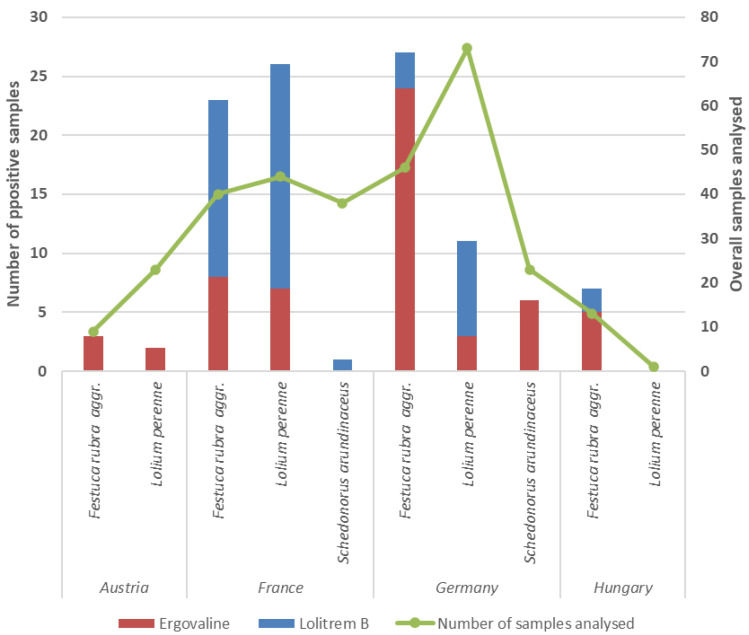
Number of positive samples for ergovaline and lolitrem B from three different grass species analyzed from grasslands of four different European countries (adapted from [11]).

Although equine endophyte toxicosis has rarely been described in Europe, multiple studies indicate that endophytes are broadly present in temperate European semi-natural grasslands. For example, Seiler et al. [11] and Khiaosa-ard et al. [12] recently monitored various meadows and pastures for horses across four different European countries (southern Germany, Austria, France, and Hungary) for the occurrence of endophyte toxins, especially ergovaline, lolitrem B, and paxilline. The authors found that around 35% of the tested samples were positive for at least one of the toxins (Figure 1), although at generally low concentrations [11]. However, ergovaline reached levels above 300 μg/kg of dry matter in 17 cases [11], with occurrence in all four countries, constituting a threat for some horse groups under certain circumstances. Interestingly, ergovaline was mainly found in samples consisting of *F. rubra* aggr. (around 37% of tested samples), independent of season, location, and grassland use (pasture or meadow) [11]; however, ergovaline was detected in samples of *L. multiflorum* (25%) and *L. perenne* (8.5%) as well [11]. *F. rubra* aggr. is a globally important turf grass and ubiquitous species in grasslands throughout Europe, found in a wide range of habitats [11]. An association between *F. rubra* and endophytic fungus *E. festucae* is described as common and is found at a high frequency in Mediterranean permanent grasslands [13]. Thus, it seems that there is a similar risk for the occurrence of *F. rubra*-related endophytes in the grasslands of central European countries as for its occurrence in Mediterranean permanent grasslands. Species with a relatively lower occurrence of ergovaline were *F. arundinaceum* (9.8%) and *F. pratensis* (3.6%) [11]. While high infestation rates (81%) of *F. pratensis* with *Epichloë* spp are reported in German grasslands [8], *F. arundinaceum* is known for infection by the endophyte of the genus *E. coenophiala*, and *L. perenne* for infection by *E. festucae var. lolii*, producing ergot alkaloids (i.e., ergovaline) responsible for fescue toxicosis in Australia, New Zealand, and the United States [1,9]. In contrast to these countries, studies in German grasslands have reported that *E. festucae var. lolii* infesting *L. perenne* often lacks dmaW gene [8], which is needed for the ergovaline synthesis, likely explaining the relatively lower ergovaline occurrence rate in *L. perenne* samples in the German grasslands, compared to other countries (Figure 1). Generally, lower occurrences (<20% of samples) and concentrations (<1100 μg/kg dry matter) were reported for lolitrem B, which was also found in most European countries but mainly in samples of *L. perenne* [11,12], a plant species which has been associated with *E. festucea var. lolii* in many countries [8,9]. In fact, lolitrem B was frequently detected in *F. rubra* as well [11], indicating that *E. festucea var. lolii* can also infest *F. rubra*.

**Figure 2:**
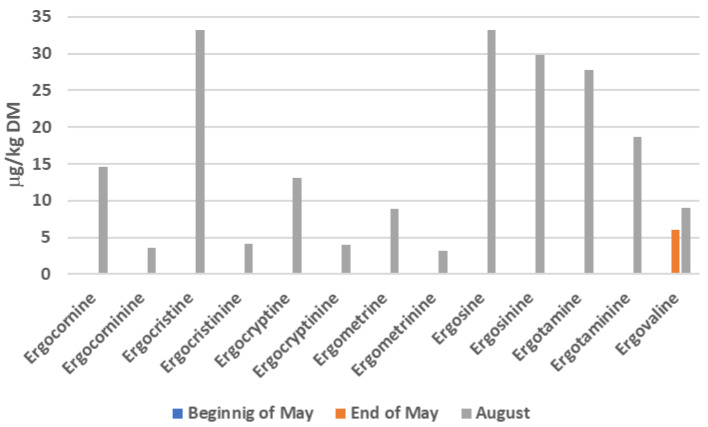
Changes in the concentration of ergot alkaloids in Austrian meadows harvested either twice during spring (at the beginning of May, there was no detectable concentration of ergot alkaloids) or in summer (adapted from [14]).

Son et al. [14] evaluated changes in the concentration of various ergot alkaloids in selected semi-extensive Austrian horse grasslands and reported co-occurrence of various ergopeptines, which increased especially during hot and arid summer months (Figure 2), indicating that the season and especially weather conditions (aridity and drought) play an important role in the occurrence of ergot alkaloids on equine pastures. Similar results have been reported in a study conducted in a perennial ryegrass field culture in southern France [15], whereby the concentrations of ergovaline varied considerably (from 526 to 2322 μg/kg of dry matter), but increased with increasing temperature.

**Figure 3:**
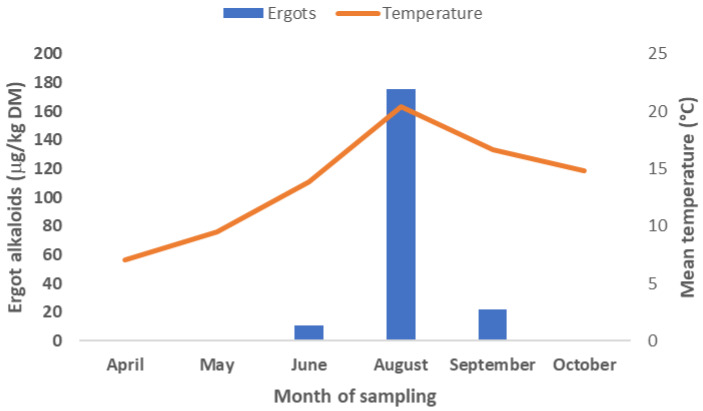
Concentration of ergot alkaloids in Austrian pastures and meadows measured during spring, summer and autumn with respective temperatures (adapted from [16]).

The same result was also found in the studies by Penagos-Tabares et al. [16], whereby the concentration of the ergot alkaloids in pastures increased during summer months with increasing temperature. During April and May, as well as October, no ergot alkaloids were detected (Figure 3). No relationship between temperature and ergovaline concentrations in pastures and meadows was reported [11]. The same research [11] reported an association between endophytes and soil humidity/rainfall, indicating that not only aridity, but also high humidity, might affect endophytes and their alkaloid concentrations.

Thus, depending on the time of year, vegetation (presence of certain plant species) and weather conditions, grasses infested with endophytes may contain higher or lower amounts of ergot alkaloids. Interestingly, other studies have indicated that not all parts of the plant have the same level of alkaloids. While growing within the grass plants, endophytes are first located at the base of the plant. As the plant grows, the fungus moves toward the growing stem until it reaches the seedheads. This ensures the dissemination of the fungus. Indeed, the toxicity is highest in the fully ripe stage, where seeds (and fungi) are in blossom [15,17]. Varying alkaloid concentrations at different maturity stages of the plants are already known [17,18]. In one study, for instance, the ergovaline content of all parts of the plant was between 300 and 500 µg/kg from March until the middle of June, but increased at the end of June, reaching levels between 1000 and 5000 µg/kg in the seedheads [17]. However, at the same time, the rest of the plant remained at the 300 to 500 µg/kg level. Penagos-Tabares et al. [16] and Son et al. [14] did not find any detectable level of ergopeptine alkaloids in Austrian horse pastures in April or at the beginning of May. However, at the end of May, ergovaline levels were found, and during July-August sampling, all ergopeptine alkaloids were measured (Figure 3). König et al. [18] measured the mean concentrations of lolitrem B and peramine in *E. festucae var. lolii*-infected *L. perenne* samples and also reported significantly higher concentrations of lolitrem B (3.4 vs. 0.5 μg/g) and peramine (2.4 vs. 0.7 μg/g) in summer vs. spring. Additionally, it seems that the ergovaline concentration increases when plants are fertilized with nitrogen [17], although a reverse effect has been reported in other studies [11].

**Figure 4:**
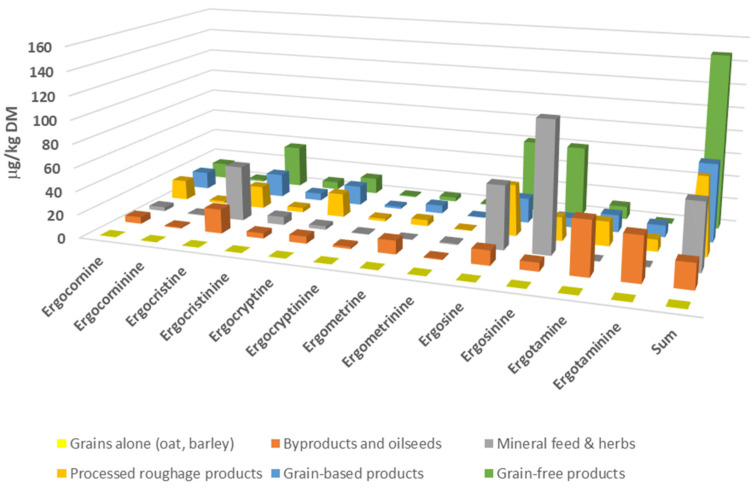
Concentration of ergopeptine alkaloids in single (grains) and various supplemental feeds for horses across various European providers (adapted from [3]).

An additional source of ergot alkaloids for horses—yet an underestimated source—is supplementary feeds, which is commonly used to supplement the equine diet. The equine market of such supplementary feeds has recently been highly diversified, and certain categories of horses, such as mares, foals, and performance horses, typically receive large amounts in their daily ration. Supplementary feeds consist of several ingredients including roughage products (hay or alfalfa cobs, cubes), grains, sugar beet and oilseed products, and various herbs. As demonstrated in Figure 4, certain supplementary feeds containing grass products such as grain-free products, either grain-free muesli or grain-free mashes may contain significantly higher concentrations of multiple ergot alkaloids (“cocktail effect”) than the other feedstuffs that do not contain grass products. Although ergovaline was not measured in that study, the sum of other ergopeptine alkaloids reached levels above 150 μg/kg in the equine grain-free feed products tested. Indeed, “cocktail effects” due to the presence of multiple toxins including ergot alkaloids have been reported in pastures [14,16], but their effects on animal health are largely understudied.

## 3. Fescue Toxicosis in Horses: Exposure Risks and Symptoms

Fescue toxicosis is a collective term for illnesses caused by tall fescue grass species infected with *E. coenophiala* that produce a variety of ergot alkaloids, of which, ergovaline is the main ergopeptine and the perpetrator of fescue toxicity syndrome [6]. These ergopeptine alkaloids affect the neuroendocrine system in horses, so that intoxication can take on different forms. The reproductive form is the most common in mares, manifested as hypoprolactinemia as a result of decreased secretion of prolactin due to ergot alkaloids binding to D2-dopamine receptors in the anterior pituitary [6,19]. Prolactin plays a key role in many physiologic processes, especially in the events surrounding birth and lactation, so that other effects of fescue toxicosis can also be observed in offspring. In addition, ergot alkaloids, such as ergovaline, also have the ability to inhibit D1-dopamine receptors, and they partially act as α1-adrenergic and serotonin receptor agonists, resulting in vasoconstriction of blood vessels [20].

Experimental evidence of vasoconstriction for ergovaline and ergovalinine was provided by McDowell et al. [21] using a Doppler ultrasound. They observed that horses on an endophyte-contaminated diet had a reduced artery lumen diameter, circumference and area. The consequences of vasoconstriction are more clearly visible in ruminants than in horses, as vasoconstriction is the cause of fescue foot, whereby cows experience lameness and gangrene, even losing the tips of their tails and ears, and may also endure secondary exungulation [6]. Even though fescue foot syndrome has not been reported in horses, there are hypotheses that vasoconstriction is the cause of fescue toxicosis and is responsible for the symptoms observed in horses as well. For instance, damage to placentas has been reported in mares that have given birth with fescue toxicosis. The damage includes edema, fibrosis, and mucoid degeneration of arteries—perhaps due to vasoconstriction [22].

Indeed, in horses, fescue toxicosis is mainly reflected in reproductive health, including changes in gestation length, dystocia, and decreased health in newborn foals. A few studies have also looked at reproductive health in stallions and sport horses. The following sections will provide a closer view of the clinical pictures of fescue toxicity syndrome in different vulnerable groups of horses.

### 3.1. Fescue Toxicosis in Mares

Due to the fact that symptoms of fescue toxicity have been most notable in pregnant mares, the effects of ergovaline on this specific group of horses have been given the most attention. Dosage, exposure length, and, most importantly, the pregnancy phase when the exposure occurs seem to affect the outcome of fescue toxicosis in mares. Table 2 summarizes results from studies on mares either between conception and the end of the first trimester of pregnancy or during the last trimester of pregnancy. Accordingly, no symptoms were apparent in mares between conception and the end of the first trimester of pregnancy that consumed up to 271 μg/kg feed of ergovaline per day. With 308 μg/kg feed consumed, weight loss and reduced serum prolactin levels were observed; however, pregnancies remained unchanged through day 28. At 867 μg ergovaline/kg feed, mares displayed decreased serum progestogen concentrations, pregnancies remained intact and fetal development was unchanged. Detrimental effects on embryo viability were observed by Brendemuehl et al. [23], who fed mares on tall fescue grass, of which 90 to 100% of plants were infected with *A. coenophialum*, with an average ergovaline concentration of 1171 μg/kg of fresh weight grass. These authors found significantly longer (+7 days) diestrus length in mares fed on ergovaline-contaminated pastures, but only a numerical difference in embryo mortality. One mare out of 13 (7.6%) from the endophyte-free group reabsorbed the embryo, whereas 6 out of 20 mares (30%) experienced an early gestational pregnancy loss associated with significantly lower blood progesterone levels.

When mares were exposed to ergovaline during the last trimester of gestation (day 250 onwards), it seems that even lower dosages of ergovaline are associated with various symptoms (Table 2). Accordingly, feeding mares on seeds with a minimal level of 200 µg ergovaline/kg of total daily ration during the last month of the pregnancy led to reduced mammary development and prolonged gestations, foal losses and agalactia [24]. Other studies have shown that concentrations of ergovaline at 300–500 μg/kg feed leads to prolonged gestation, dystocia, agalactia, high foal mortality, retained placentas, and very little udder development [22,25]. This level is also close to recommendations released by the Oregon State University Endophyte Service Laboratory [26,27] stating that mares should not consume more than 300–500 μg/kg of ergovaline per day. These guidance levels of ergovaline should be interpreted with caution because their toxicity depends on many factors, including physiological status, individual sensitivity, overall feed intake, and likely other factors that might affect resorption and metabolism of the alkaloid.

Monroe et al. [28] used 16 mares grazing either on endophyte-free pastures throughout their gravidity or on endophyte-infested pastures (mainly *E. typhina*). The study found that foals from mares grazing on endophyte-infested pastures tended to be on average 4 kg heavier than those in the endophyte-free group. This can be explained by the longer gestation period (+27 days) of the mares. Other sources have reported dystocia to be another result of fescue toxicosis in horses [29], which again can be explained by the lengthened gestation period, as heavier and larger foals are more difficult to birth, increasing the odds for retained placenta and uterine infections, which in turn may increase the risks for infertility and laminitis. Abortion and stillbirths are also symptoms of fescue toxicosis, albeit not considered typical [30].

Since fescue toxicosis commonly results in significantly lower serum prolactin, Evans et al. [6] suggested that there may be a link between prolonged gestation and hypoprolactinemia. Low serum prolactin levels in the mare may induce changes in the uteroplacental pathways that lead to post-term delivery of the fetus [25]. Low serum prolactin levels lead to decreased milk production or even agalactia in pregnant mares, especially toward the end of gravidity [30]. There were also observations that mares removed from endophyte-infected pastures one month prior to parturition recover well from fescue toxicity and deliver typical foals, although their milk production may still be decreased [25]. It seems that fescue toxicosis is somehow reversible to a certain degree if the exposure does not occur during the last month of gestation. On the other hand, the fescue toxicosis during the last month of pregnancy may have noticeable effects on neonates, as shown below.

### 3.2. Fescue Toxicosis in Offspring and Stallions

Reproductive health disorders of fescue toxicosis may not be limited only to mares; they may also result in health and developmental issues in their offspring. Not only do mares exposed to endophyte alkaloids throughout their gravidity have a higher chance of delivering stillborn foals, but the born foals are also more likely to be weak, either dysmature or overmature (or show signs of both), and later display signs of inhibited growth [2]. The physical appearance of foals born from mares exposed to endophytes has been described as large with spindly extremities; long and fine haircoats; overgrown hooves, bare from the protective eponychium; and lacking in muscle mass, with central incisors likely erupted [30]. Research has also shown that weak foals may have a poor suckling reflex, with a lower ability to absorb IgG due to a failure of passive transfer [24,30], which can be life-threatening to foals as they are born without antibodies. Failure of passive transfer may lead to septicemia, a serious condition, which is also a known symptom of fescue toxicosis in mares when they suffer from retained placentas. Consequently, foals born to mares that have grazed ergot alkaloid-infested pastures have a more difficult start in life and may need veterinary assistance, which is related to high costs, and the prognosis in cases of septicemia is uncertain.

When compared with mares, little is known about the effects of alkaloid toxicity in stallions, which seemingly do not show any apparent symptoms. The study conducted by Fayrer-Hosken [31] examined six stallions that were fed either endophyte-positive fescue seeds or not and monitored semen regularly over one breeding season. The authors found that stallions from the alkaloid-positive group had a lower volume of gel-free ejaculate, but otherwise there were no differences in the spermiograms. Sperm motility, sperm concentration, sperm cell morphology, testicular morphology and temperature, hormone levels, and the number of straws for artificial insemination were the same. In another study, Fayrer-Hosken et al. [32] studied 1.5-year-old colts that were fed endophyte-positive seeds over the entire course of a spermatogenic cycle of 70 days and found that toxic fescue seeds did not affect spermatogenesis. The ability to breed was not impaired, and ergot alkaloid-infested feed did not affect the testes of colts on a cellular basis [32]. Unfortunately, none of the studies provided information about the level of ergot alkaloids in the feed. Therefore, it is difficult to derive an exposure threshold and establish a dose–response relationship for stallions.

### 3.3. Fescue Toxicosis in Exercising Horses

Exercising horses are exposed to additional stress during their daily training sessions, so potentially adverse vasoactive effects of ergopeptine alkaloids [33] in the diet on their cardiovascular and respiratory systems can be expected. One of the first studies was conducted in 14 horses, either Arabians or Quarter Horses, which were randomly divided to graze either on pasture with endophyte-positive fescue or on a pasture that was devoid of endophytes [34]. The horses received no other feed and remained in their respective pastures for two weeks. Two horses, one of each group, were exercised together, and their vital parameters were measured immediately before exercise and a total of eight times post-training. While no differences were observed prior to exercise, horses from the endophyte-positive group had higher respiratory rates than horses from the control group over the course of 210 min of training. Horses from the endophyte-free group were back to their pre-exercise heart rates after 90 min, as opposed to horses from the endophyte-positive group, which only reached pre-exercise heart rates after 150 min. Skin temperature was back to normal for horses in the endophyte-free group after 90 min; in the endophyte-positive group it took 180 min to lower skin temperature to the same degree as before exercise. Thus, endophytes in the diet negatively impacted exercising horses, likely due to vasoconstriction, which impaired heat dissipation, leading to higher core temperatures, prolonged recovery times, increased respiration rates, and reduced blood flow to skin and core tissues, impairing performance by hindering the body’s cooling mechanisms. This might be a particular issue in hot/humid exercising conditions. Although the heart rate response was less affected in that study, the horses that were fed alkaloid-infested grass had a higher intake of water compared to the control group [34], likely due to hyperthermia. Table 3 gives an overview of the effects of different exposure rates of ergot alkaloids on health parameters in exercising horses.

Another study was conducted in 12 horses fed fescue hay that contained high levels of ergot alkaloids, averaging 1995 μg/kg [35]. On day 14 of the test period, horses were subjected to an exercise tolerance test consisting of 4 min walk, 14 min trot, and 6 min lope/gallop, with target heart rate ranges of 50 to 70, 71 to 110, and 111 to 150 beats per minute, respectively. Increased excretion of ergot alkaloids was observed in urine samples. However, no increase was observed in pre-exercise or post-exercise rectal temperatures or pulse or respiration rates until after 10 min of recovery, indicating that high dosage of ergot alkaloids did not affect post-exercise recovery when horses were subjected to a light workload. Another study evaluated 10 Quarter Horses that were fed tall fescue seed containing 459 μg/kg of ergovaline [36]. On several days, horses were subjected to a standardized exercise test that was designed to raise their heart rate beyond the anaerobic threshold (150 beats per minute). Although there was no effect of ergovaline on heart rate, post-exercise whole blood lactate level, and rectal temperature, consumption of diets that averaged 450 ppb of ergovaline caused the horses to expend more respiratory effort in an attempt to recover to a resting rectal temperature. Another study evaluated 10 horses that were fed fescue seed that contained both ergovaline and ergotamine (altogether 406 ppb) [37]. The aerobic test consisted of walking, trotting, and loping and was designed to maintain the horse’s heart rate at less than 150 beats per minute. The anaerobic test was designed to increase the horse’s heart rate to more than 150 bpm. In this study, there were no treatment effects on water consumption, sweat production, or rectal temperature at rest or during recovery from the anaerobic exercise. However, rectal temperatures and respiration rates increased after the aerobic exercise, and respiration rates were higher after 30 and 60 min of the anaerobic exercise for horses consuming ergot alkaloids, likely to compensate for a reduction in the efficiency of evaporative cooling, which resulted from vasoconstriction of peripheral blood vessels.

The evidence suggests that the intake of ergot alkaloids has consequences for exercising horses, but it seems to depend on the exposure dosage and the type of exercise, whereby the effects seem to be stronger, the higher the exercise intensity. Due to their vasoconstrictive characteristics, ergot alkaloids seem to affect thermoregulation and prolong recovery after exercise. This not only puts a higher physical demand than necessary on horses’ bodies and cardiovascular and respiratory systems, but it can also have a negative impact on sport horses that compete in eventing and endurance racing, where vital parameters are adduced as indicators of whether a horse is fit enough to continue competing or needs to be taken out.

## 4. Equine Fescue Edema

Equine fescue edema falls under the umbrella term “fescue toxicosis”, as this syndrome is caused by the consumption of tall fescue grass. Although the exact causal agent has not yet been defined, it is believed that endophytes (*E. coenophiala*) that grow within tall fescue grass, probably a compound of the subclass of lolines—N-acetyl norloline—might play a role [9], although this was not confirmed in another study [38]. Equine fescue edema has so far only been reported in Australia and New Zealand, with the first cases affecting 56 horses across Australia between the fall of 2007 and 2008 [9]. These authors collected cases of horses that suffered from a sudden onset of lethargy, inappetence, and presented subcutaneous swelling in various areas of the body, including the eyelids, lips and the neck. Subcutaneous edema of the abdomen and the vulva were also reported, and one horse had a rectum prolapse. Blood analysis showed lowered serum protein levels, especially albumin. In one case, four out of five mares and three of their newborn foals presented with these symptoms while grazing on pasture. They were removed from the pasture and eventually made a full recovery. Nevertheless, the four affected mares decreased in bodily fitness, and their foals lagged in growth. The three sick foals remained considerably smaller than the norm. Noteworthy, too, is the fact that the equine fescue edema seems to have impacted these mares’ reproductive health. Some mares showed edematous uteruses, and they did not come into heat for six weeks. When they did, it took many cycles of artificial insemination before they became pregnant [9]. In another case, horses presented in a similar way. However, one death was reported. On this farm, sheep were exchanged for the horses, and they did not fall ill after eating the alkaloid-infected tall fescue. Similarly, in a different case, cattle were put on the pasture after horses became ill, and they were also not affected [9]. What is noteworthy in Bourke et al.’s case report is that they noticed a similarity in the weather conditions prior to the horses becoming sick. In multiple cases, a 4- to 6-week-long drought was followed by heavy rainfall, which induced massive plant growth [9] and, likely, endophyte growth and increased concentrations of alkaloids.

Munday et al. [39] investigated the pathologies caused by equine fescue edema induced by grazing Mediterranean tall fescue (*L. arundinaceum*) infected with *E. coenophiala*. Five horses were put on infected pastures. As soon as they showed clinical signs of the syndrome, blood was drawn, they were euthanized, and an autopsy was performed. The bloodwork showed significantly lowered levels of total protein and increased packed cell volume. The findings of the autopsy included submucosal edema of the duodenum, small intestine, caecum, colon, and rectum, and about 2 L of clear liquid ascites. Lymph nodes of the colon and caecum were enlarged. In addition, a large number of eosinophils were found in the intestine, perhaps due to heightened vascular permeability. In a subsequent study, Finch et al. [38] experimented with the same set-up as before and tried treating affected horses with 250 mg of the corticosteroid methylprednisolone and 300 mg of the anti-histamine cetirizine hydrochloride orally every 12 h for a 7-day period before they were euthanized and necropsied. The intent was to compare the findings of the autopsy to the findings from the previous study. The medication did not prevent the onset of equine fescue edema, and it also did not reduce the severity of the symptoms. By day 7, all horses developed signs of fescue edema, and concentrations of total protein in the serum decreased (<45 g/L) compared with healthy horses (>60 g/L). Necropsy showed marked edema and eosinophilic inflammation in the intestines of all horses grazing Mediterranean tall fescue.

## 5. Ryegrass Staggers

Ryegrass staggers is a sickness that has been described in horses grazing on perennial ryegrass (*L. perenne*) infested with *E. festucae var. lolii*. The fungus can be found in two places on the grass plant: at the bottom of the leaf sheath and in the infructescence of the reproductive saplings [16]. It produces lolitrem B, which is a known tremorgen that affects the nervous system and indirectly the muscular system. Ryegrass staggers is only associated with perennial ryegrass [40], although there is recent evidence that other grass species such as *F. rubra* aggr. seem to produce lolitrem B, even though at low concentrations [11].

Animal species other than equines reportedly affected by ryegrass staggers include sheep, cattle, deer, goats, llamas, camels, alpacas, and rhinoceroses [2]. Many reports have described intoxicated sheep and cattle, especially in New Zealand and Australia. Little is known about the incidence rate on the European continent. However, sporadic incidents have been documented in Germany, France, the Netherlands and the UK [2,41]. One reason for the frequent outbreaks in Australia and New Zealand could be that both countries tend to grow monocultures on their grasslands, and if these are infested with toxic fungi, grazing livestock will ultimately consume larger amounts of toxins [40]. In comparison, European forages tend to display a variety of grass plant species, and if one species is infected, it only makes up a fraction of daily feed intake [2,11,18,41].

In horses, symptoms of ryegrass staggers include muscle weakness, tremors, and spasms. The clinical picture is especially noticeable in horses going at a fast pace, e.g., trotting or galloping, where they may show signs of trembling and ataxia and threaten to fall [42]. These neurogenic symptoms may also occur when high amounts of the toxin have accumulated in the body, whereas the first symptoms of lolitrem B intoxication may be a reduction in daily weight gain and milk production.

The clinical picture of ryegrass staggers was experimentally described by Johnston et al. [42], who fed 7 horses, first with endophyte-free hay for 1 week, followed by ryegrass contaminated with lolitrem B-producing endophytes for 2 weeks. Their total ration contained 2 mg lolitrem B per day, which was the threshold established to display neurological signs in bovines [28]. Their findings were that horses showed the onset of clinical symptoms 4 to 9 days after initial ingestion of lolitrem B, and that the severity of symptoms varied between horses but was not related to breed, age, sex, or body weight. Horses showed tremors, mainly of the forehand, that worsened when being fed or lunged. Additionally, horses displayed signs of ataxia, including truncal sway, wide placement of the limbs when walking, stumbling, toe dragging, irregular gait, and threatening to fall when asked to do more difficult exercises. The horses were also overexcited and reacted more heavily to threatening gestures than was typical. Additional findings were an increase in heart rate while on a lolitrem B-positive diet as opposed to before the trial. Two horses also suffered from edema of the legs, which occurred nine days after the test period started. The symptoms disappeared after the trial when the horses were being fed endophyte-free hay. However, no clinical signs remained in the long term [42].

Studies conducted in Europe (Germany, France, Austria, and Hungary) indicate the non-ubiquitous occurrence of lolitrem B (and paxilline—a precursor of lolitrem B) in pastures [8,11,12,15,18], although the overall concentration was considered to be rather low to cause acute toxicity [11,12]. However, as much as 14% of the samples collected from three sites in Germany exceeded the toxicity thresholds set for ruminants [18], and endophyte-infected perennial ryegrass inflorescence samples collected at the fully ripe stage in France also exceeded this threshold [15]. Although the risk for ryegrass staggers seems to still be rather low in Europe, especially when grasslands are not dominated by *L. perenne*, regular monitoring and control of seeds and grasslands is required to avoid intoxication, especially in light of climate warming, which, as shown in some studies [12,15,18], is associated with greater lolitrem B concentrations. 

## 6. Treatment and Preventative Measures

The main prevention strategy against equine fescue toxicity in horses is based on avoiding endophyte-positive grasslands. As described above, the main challenge is that concentrations of the alkaloids can change, being affected by several vegetation and geo-climatic factors. There are laboratories that offer screenings of feed samples for endophyte alkaloids. However, laboratory testing is not widespread, and sometimes horses cannot be easily resettled to endophyte-free grasslands. Hence, research has looked at several other preventative and treatment tools against equine fescue toxicosis, such as the administration of D2-dopamine antagonists, aiming to reverse the effects of ergovaline on D2-dopamine receptor cells and inhibit the release of prolactin. Such antagonists tested have been domperidone and sulpiride. Redmond et al. [43] conducted a study on sixteen pregnant mares (Quarter Horses or Arabians) fed ergovaline-infected hay starting from 30 days before the assumed term. Mares were initially given 0.55 mg/kg of domperidone or 1.65 mg/kg of sulpiride orally every day. After the first mare in each group gave birth, it was noticed that the dosages seemed to be too low because there was a lack of udder in both mares, so the dosages were doubled. From then on, mares either received 1.1 mg/kg of domperidone once a day or 3.3 mg/kg of sulpiride. Some changes were also made in the three groups, and in the end, the control group consisted of 4 horses, the domperidone group of 5, and the sulpiride group of 4 animals. Notably, in both treatment groups all foals were vital, whereas in the control group, only 3 out of 4 foals lived. In both treatment groups, foals were a little bit lighter than in the control group. All foals were proportional in size and muscularity. They showed neither signs of prematurity nor overmaturity.

One major difference between the treatment groups and the control group was in the length of total gestation. The control mares gave birth after approximately 350 days of gestation, which was 11 days overdue. In contrast, the domperidone group had an average gestation length of 338 days. Sulpiride also had an effect on gestation length, although not as strong as domperidone, but gestation length was at around 343 days for the mares in this group, and they birthed their foals roughly 3 days after the expected date of delivery. There was also an improvement in terms of agalactiae, as none of the mares from the domperidone and sulpiride groups suffered from the lack of milk. However, there were unexpected findings regarding placenta retention and conception rates. Perhaps this was due to the small size of the study, but in both the domperidone group and the sulpiride group there was one mare that experienced a retained placenta, while in the control group, no mare was affected. In addition, all mares from the control group conceived the first time they were bred following parturition. The mares from the treatment groups, on the other hand, had lower conception rates. Two out of five mares in the domperidone group, and only half of the mares in the sulpiride group were successfully bred [43]. Positive changes were observed in udder growth after 9 days on domperidone and after 14 days on sulpiride. The control group on the endophyte-infested pasture did not present any changes in udder development until they were removed from the pasture. Prolactin serum levels rose after 4 days for the domperidone group and after 9 days for the sulpiride group and were high at parturition.

Cross et al. [24] also conducted a study on the efficacy of oral administration of domperidone (1.1 mg/kg) 15 days prior to the expected foaling date on parturition and agalactiae on mares that were fed an ergovaline-positive diet (200 μg/kg diet). Domperidone was able to shorten gestation length and maintained the amounts of milk at three and five days after foaling, preventing agalactia. However, although only 1 out of 13 mares did not successfully respond to the domperidone with treatment in terms of gestation length, mammary development, and milk production, some mares experienced dripped milk before foaling, and almost all foals suffered from failure of passive transfer, suggesting that domperidone may not undo all the consequences of endophyte toxicosis in mares.

Another strategy to deal with endophyte toxicosis has been suggested to be the use of endophyte-free or novel nontoxic ergot alkaloid-producing endophyte lines. Novel endophytes are specifically inserted into tall fescue and perennial ryegrass, generating cultivars that do not produce ergot and lolitrem alkaloids while still being toxic to insects and protecting the plants from insect damage, which is an added benefit [44]. Seeds of novel endophyte tall fescue are available for purchase in New Zealand, Australia, and the USA.

McDowell et al. [29] conducted a two-year study to test the efficacy of novel endophytes in pregnant mares. Six late-gestational mares were grazing on a pasture with novel endophyte tall fescue (NETF). For comparison, 6 late-gestational mares were put on a pasture with orchardgrass, a different grass species, devoid of the endophytes that produce ergovaline. In addition, four nonpregnant mares grazed a tall fescue pasture that was infected with a strain of ergot alkaloid-producing *Epichlöe*. The aim of the study was to compare mares grazing on novel endophytes with mares grazing on zero endophytes and, additionally, to compare the sizes of the palmar artery of mares from novel endophyte pastures and mares from ergovaline-infected pastures. This experimental set-up was repeated for a second year. In addition to palmar artery size, the authors looked at serum estradiol, progesterone, and prolactin concentrations; placental weights; placental lesions; foal viability; retained placentas; udder development; growth rate in foals; and the nursing behavior of foals. Their study corroborated that the use of NETF is a valuable method to prevent fescue toxicosis syndrome, as the outcome is that there is no significant difference in any of the observed items between foaling mares on NETF pastures and those on orchardgrass pastures. There were no retained placentas or dystocias, all foals were viable and well-developed, foals grew at regular rates and nursed well from the beginning, mares’ mean hormone levels were the same in both groups, and there was no difference in the diameter of the palmar artery lumen. Although the use of novel endophyte fescue promises good results against endophyte toxicosis, substantially higher seed costs [45] and other regulatory issues are still a burden to resolve for their use in practical terms. In conclusion, preventative and treatment measures employed in mares include administration of D2-dopamine antagonists to reverse the effects of ergovaline and, in some countries, the use of the novel toxin-free endophytes, whose practical use is short-term and/or costly.

## 7. Conclusions

This article provides an overview of endophyte alkaloids in grasslands and products thereof, highlighting the role of exposure levels in toxicity risks for horses kept on pasture and fed hay and other forage products. It is obvious that toxicity risk for horses depends on the exposure level and length, as well as individual animal factors such as overall feed intake, especially “cocktail effects” through intake of multiple alkaloids and the physiological status of the animal, whereby breeding, newborn and exercising horses are more susceptible to fescue toxicity. The majority of the studies suggest that the risks of endophyte occurrence and high levels of alkaloids vary depending on vegetation (i.e., plant species), plant maturity and part, and other geo-climatic conditions (especially aridity and drought) of grasslands. For European grasslands, the presence of the grass species *F. rubra*, *F. arundinacea*, or *L. perenne* seems to increase the risk for ergovaline, and *L. perenne* and *F. rubra* for lolitrem B. The paper concludes that besides agronomic (seeds, multiple plants instead of monocultures, soil characteristics) and technical (avoiding the high plant maturity stage at harvesting or grazing) measures, routine control and analysis of grasslands are needed to prevent endophyte toxicity, especially when the risk factors mentioned above are present. More research is needed to evaluate the “cocktail effects” of multiple endophyte alkaloids and other toxins including mycotoxins and other plant toxins on horse health.

## Figures and Tables

**Table 1:** Endophytes in selected grass species with alkaloids and diseases caused in horses (adapted from [1,9,11,12,13,14,15,16]).

Grass Common Name	Latin Name	*Epichloë* Endophyte	Alkaloid	Illness/Symptoms in Horses
Tall fescue Red fescue	*Festuca arundinaceum* (=*Schedonorus arundinaceus*, =*Lolium arundinaceum*) *F. rubra*	*E. coenophiala* *E. festucae*	Ergovaline, Lolines—N-acetyl norloline	Fescue toxicosis/fescue toxicity syndrome, equine fescue edema
Perennial ryegrass	*Lolium perenne*	*E. festucae var. lolii*	Lolitrem B	Ryegrass staggers
Perennial ryegrass Red fescue	*L. perenne* *F. rubra*	*E. festucae var. lolii x E. typhinae*	Ergovaline	Ergot alkaloid toxicity
Perennial ryegrass Red fescue	*L. perenne* *F. rubra*	*E. festucae var. lolii*	Paxilline	Tremorgenic symptoms

**Table 2:** Summary of the expected symptoms from ergovaline exposure in pregnant mares during various phases of gestation (adapted from [24,25]).

Concentration of Ergovaline (µg/kg) in Feed Consumed	Symptoms
Between conception and the end of the first trimester of pregnancy	
45–271	no symptoms reported
308	Weight loss, reduced serum prolactin levels, no negative effects on pregnancy through day 28
867	Decreased progestogen concentration, no effect on embryonic development, no losses of pregnancy
1171	Significantly prolonged luteal phase, decreased 14-day viable pregnancy rate per cycle, increased number of early embryonic deaths
During the last trimester of pregnancy	
200	Reduced mammary development, prolonged gestations, foal losses, agalactia
300–500	Prolonged gestation, dystocia, agalactia, high foal mortality, retained placentas, and very little udder development

**Table 3:** Summary of the expected symptoms observed from ergot alkaloid exposure in exercising horses with different levels of exercise intensity (adapted from [35,36,37]).

Concentration of Ergot Alkaloids (µg/kg) in Feed Consumed	Symptoms
Aerobic exercise (<150 beat per minute)	
406	rectal temperatures and respiration rates increased after the aerobic exercise
1995	increased excretion of ergot alkaloids in urine; no effects on pre-exercise or post-exercise rectal temperatures or pulse or respiration rates
Anaerobic exercise (>150 beat per minute)	
406	no effects on water consumption or sweat production, rectal temperature at rest or during recovery from the anaerobic exercise. However, respiration rates increased after the anaerobic exercise
459	no effect on heart rate, post-exercise whole blood lactate level, and rectal temperature; however, higher respiratory rate in an attempt to recover to resting rectal temperature

## Data Availability

No new data were created or analyzed in this study.
